# Generalizable Machine Learning in Neuroscience Using Graph Neural Networks

**DOI:** 10.3389/frai.2021.618372

**Published:** 2021-02-23

**Authors:** Paul Y. Wang, Sandalika Sapra, Vivek Kurien George, Gabriel A. Silva

**Affiliations:** ^1^ Center for Engineered Natural Intelligence, University of California San Diego, La Jolla, CA, United States; ^2^ Department of Physics, University of California San Diego, La Jolla, CA, United States; ^3^ Department of Electrical and Computer Engineering, University of California San Diego, La Jolla, CA, United States; ^4^ Department of Bioengineering, University of California San Diego, La Jolla, CA, United States; ^5^ Department of Neurosciences, University of California San Diego, La Jolla, CA, United States

**Keywords:** calcium imaging, graph neural network, deep learning, *C elegans*, motor action classification

## Abstract

Although a number of studies have explored deep learning in neuroscience, the application of these algorithms to neural systems on a microscopic scale, i.e. parameters relevant to lower scales of organization, remains relatively novel. Motivated by advances in whole-brain imaging, we examined the performance of deep learning models on microscopic neural dynamics and resulting emergent behaviors using calcium imaging data from the nematode *C. elegans*. As one of the only species for which neuron-level dynamics can be recorded, *C. elegans* serves as the ideal organism for designing and testing models bridging recent advances in deep learning and established concepts in neuroscience. We show that neural networks perform remarkably well on both neuron-level dynamics prediction and behavioral state classification. In addition, we compared the performance of structure agnostic neural networks and graph neural networks to investigate if graph structure can be exploited as a favourable inductive bias. To perform this experiment, we designed a graph neural network which explicitly infers relations between neurons from neural activity and leverages the inferred graph structure during computations. In our experiments, we found that graph neural networks generally outperformed structure agnostic models and excel in generalization on unseen organisms, implying a potential path to generalizable machine learning in neuroscience.

## 1 Introduction

Constructing generalizable models in neuroscience poses a significant challenge because systems in neuroscience are typically complex in the sense that dynamical systems composed of numerous components collectively participate to produce emergent behaviors. Analyzing these systems can be difficult because they tend to be highly non-linear in how they interact, can exhibit chaotic behaviors and are high-dimensional by definition. As such, indistinguishable macroscopic states can arise from numerous unique combinations of microscopic parameters i.e., parameters relevant to lower scales of organization. Thus, bottom-up approaches to modeling neural systems often fail since a large number of microscopic configurations can lead to the same observables (Golowasch et al. (2002); Prinz et al. (2004)).

Because neural systems are highly degenerate and complex, their analysis is not amenable to many conventional algorithms. For example, observed correlations between individual neurons and behavioral states of an organism may not generalize to other organisms or even to repeated trials in the same individual (Frégnac (2017); Churchland et al. (2010); Goldman et al. (2001)). Hence, individual variability of neural dynamics remains poorly understood and a fundamental obstacle to model development as evaluation on unseen individuals often leads to subpar results. Nevertheless, neural systems exhibit universal behavior: organisms behave similarly. Motivated by the need for robust and generalizable analytical techniques, researchers recently applied tools from dynamical systems analysis to simple organisms in hopes of discovering a universal organizational principle underlying behavior. These studies, made possible by advances in whole-brain imaging, reveal that neural dynamics live on low-dimensional manifolds which map to behavioral states [Prevedel et al. (2014); Kato et al. (2015)]. This discovery implies that although microscopic neural dynamics differ between organisms, a macroscopic/global universal framework may enable generalizable algorithms in neuroscience. Nevertheless, the need for significant hand-engineered feature extraction in these studies underscores the potential of deep learning models for scalable analysis of neural dynamics.

In this work, we examine the performance and generalizability of deep learning models applied to the neural activity of *C. elegans* (round worm/nematode). In particular, *C. elegans* is a canonical species for investigating microscopic neural dynamics because it remains the only organism whose connectome (the mapping of all 302 neurons and their synaptic connections) is completely known and well studied [White et al. (1986); Bargmann and Marder (2013); Varshney et al. (2011); Cook et al. (2019)]. Furthermore, the transparent body of these worms allows for calcium imaging of whole brain neural activity which remains the only imaging technique capable of spatially resolving the dynamics of individual neurons (Wen and Kimura, 2020). Leveraging these characteristics and insight gained from previous studies, we developed deep learning models that bridge recent advances in neuroscience and deep learning. Specifically, we first demonstrate state-of-the-art performance for classifying motor action states-e.g., forward and reverse crawling-of *C. elegans* from calcium imaging data acquired in previous works. Next, we examine the generalization performance of our deep learning models on unseen worms both within the same study and in worms from a separate study published years later. We then show that graph neural networks exhibit a favourable inductive bias for analyzing both higher-order function and microscopic/neuron-level dynamics in *C. elegans*.

## 2 Background

In this section we discuss recent advances in neuroscience and machine learning upon which we build our model and experiments.

### 2.1 Universality/Generalizability in Neuroscience

The motor action sequence of *C. elegans* is one of the only systems for which experiments on whole-brain microscopic neural activity may be performed and readily analyzed. As such, numerous efforts have focused on building models that can accurately capture the hierarchical nature of neural dynamics and resulting locomotive behaviors [Sarma et al. (2018); Gleeson et al. (2018)]. Taking advantage of this, Kato et al. (2015) investigated neural dynamics corresponding to a pirouette, a motor action sequence in which worms switch from forward to backward crawling, turn, and then continue forward crawling. Their analysis showed that most variations (∼65%) in neural dynamics can be expressed by three components found through principal component analysis (PCA) and that neural dynamics in the resulting latent space trace cyclical trajectories on well-defined low dimensional manifolds corresponding to the motor action sequence (Supplementary Figure S1). By identifying individual neurons, an experimental feat, these authors further determined that these topological structures in latent space were universally found among all five worms imaged in their study.

**FIGURE 1:**
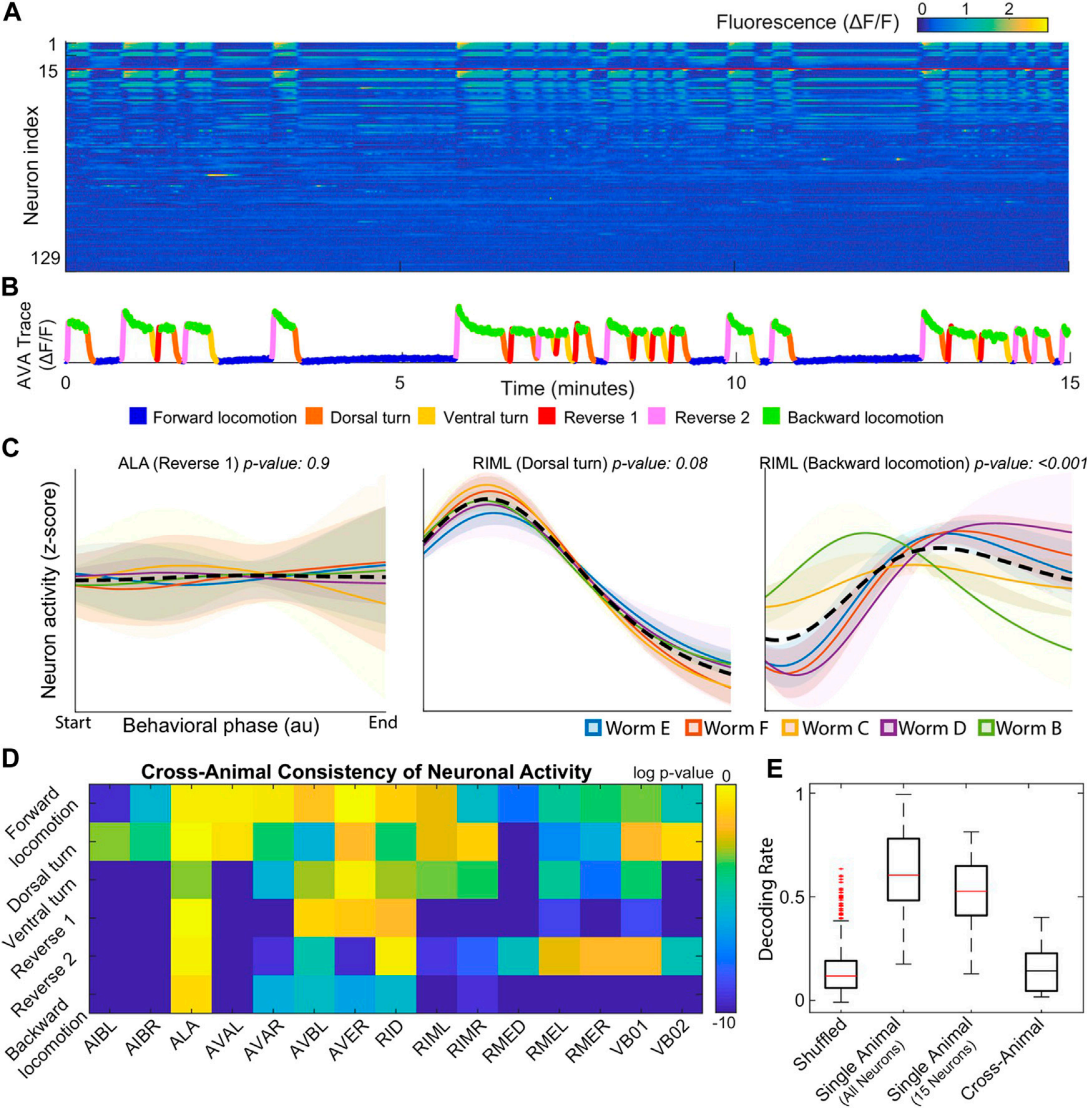
**(A)** Calcium signals recorded in one animal for ∼15 min by Kato et al. (2015). Each row represents a single neuron. The top 15 rows (above the red line) correspond to neurons unambiguously identified in all animals (shared neurons) **(B)** Sample trace with corresponding behavioral state colored **(C)** Neural dynamics of two neurons for specific behavior states. Colored solid lines are the mean activity for each animal, and the black dashed line is the mean activity for all animals. Shaded colored regions show 95% confidence intervals **(D)** Probabilities that neural dynamics from different individuals were drawn from the same distribution **(E)** Attempt by Brennan and Proekt (2019) to decode onset of backwards locomotion using neural dynamics for each animal and averaged neural dynamics across other four animals. Reproduced with permission from Brennan and Proekt (2019).

Following Kato et al. (2015), the authors published several studies focusing on global organizational principles of C. Elegant behavior [Nichols et al. (2017); Kaplan et al. (2020); Skora et al. (2018)]. Building on two of these works, Brennan and Proekt (2019) found consistent differences between each individual’s neural dynamics, precluding the use of established dimensional reduction techniques. For example, among 15 neurons uniquely identified among all 5 worms, only 3 neurons displayed statistically consistent behavior (Figure 1D). Examples of inconsistent behavior for unequivocally identified neurons (ALA and RIML) are shown in Figure 1C where the average of ALA’s activity fails to resemble the behavior of any worm and where RIML’s activity is consistent among all animals during dorsal turns, but inconsistent during reverse crawling. Resulting from these discrepancies, topological structures identified by performing PCA on each worm’s neural activity were no longer observed when data from all worms was pooled together.

**FIGURE 2:**
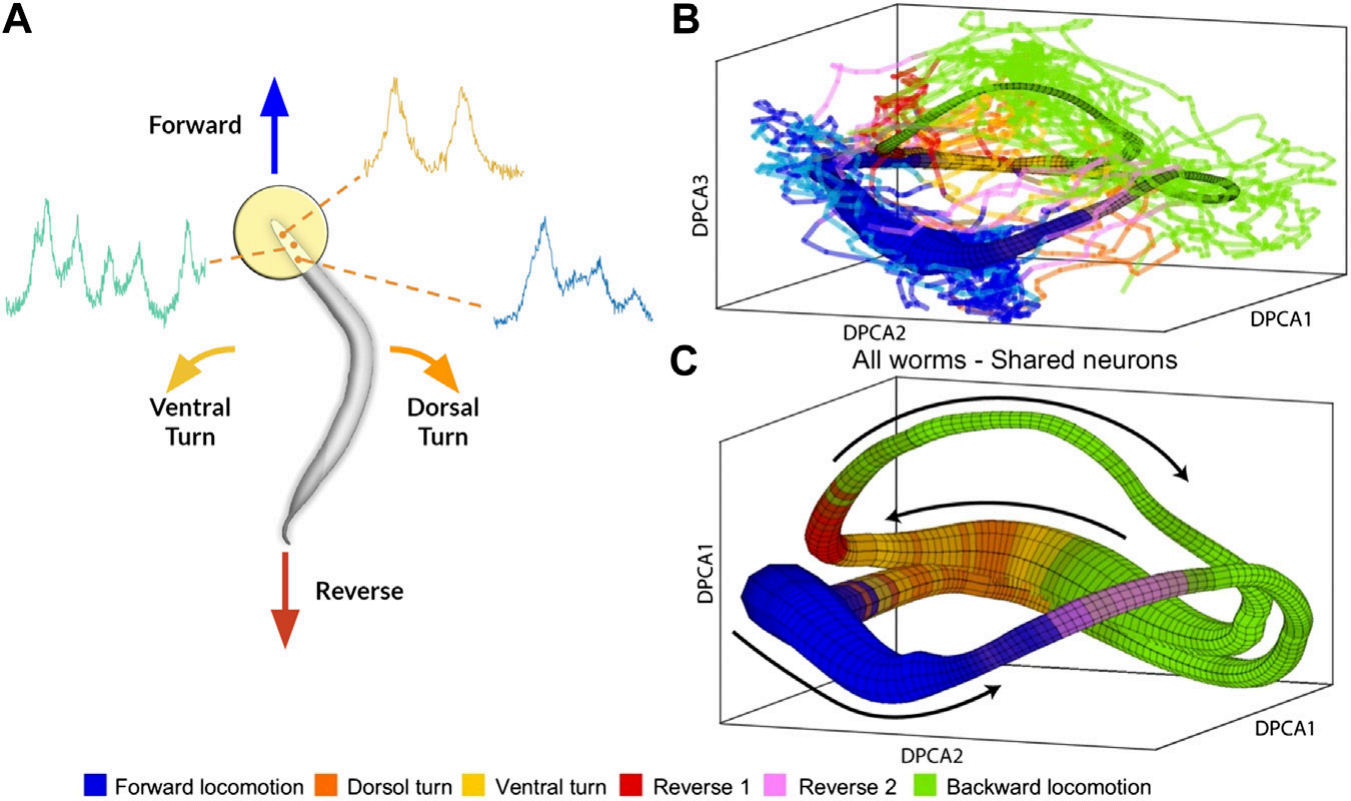
**(A)** Rendering of calcium imaging experiment where activity of neurons in the head of the worm is recorded. Colored arrows show main motor action behavioral states **(B)** and **(C)** Resulting manifold from Brennan and Proekt (2019) **(B)** Manifold constructed from activity of four worms with colored lines indicating neural activity of fifth worm **(C)** Manifold constructed from neural activity of uniquely identified neurons (*n* = 15) shared among all five worms. Black arrows correspond to cyclical transition of motor action sequence and colors correspond to motor action states. Modified with permission from Brennan and Proekt (2019).

To address this issue, Brennan and Proekt (2019) introduced a new algorithm, Asymmetric Diffusion Map Modeling (ADMM), which maps the neural activity of any worm to an universal manifold (Figure 2). To achieve this, ADMM first performs time-delay embedding of neural activity into phase space. Next, a transition probability matrix is constructed by calculating distances between points in phase space using a Gaussian kernel centered on the subsequent timestep. Finally, this asymmetric diffusion map is used to construct a manifold representative of neural activity. Contrasting conventional dimensional reduction techniques, ADMM allowed quantitative modeling by mapping neural activity from the manifold, and enabled the prediction of motor action states up to 30s ahead. Despite its success, the algorithm heavily relies on hyperparameters, such as embedding parameters, which are difficult to justify and tune.

### 2.2 Graph Neural Networks

Graph Neural Networks (GNNs) are a class of neural networks that explicitly use graph structure during computations through message passing algorithms where features are passed along edges between nodes and then aggregated for each node [Scarselli et al. (2009); Gilmer et al. (2017)]. These networks were inspired by the success of convolutional neural networks in the domain of two-dimensional image processing and failures when extending conventional convolutional networks to non-euclidean domains Battaglia et al. (2018). In essence, because graphs can have arbitrary structure, the inductive bias of convolutional neural networks [equivariance to translational transformations (Cohen and Welling, 2016)] often breaks down when applied to graphs. Addressing this issue, an early work on GNNs showed that one-hop message passing approximates spectral convolutions on graphs [Kipf and Welling (2016)]. Subsequent works have examined the representational power of GNNs in relation to the Weisfeiler-Lehman isomorphism test Xu et al. (2018) and limitations of GNNs when learning graph moments [Dehmamy et al. (2019)]. From an applied perspective, GNNs have been widely successful in a wide variety of domains including relational inference [Kipf et al. (2018); Löwe et al. (2020); Raposo et al. (2017)], node classification Kipf and Welling (2016) Hamilton et al. (2017), point cloud segmentation (Wang et al., 2019), and traffic forecasting Yu et al. (2018); Li et al. (2018).

### 2.3 Relational Inference

Relational inference remains a longstanding challenge with early works in neuroscience seeking to quantify correlations between neurons Granger (1969). Modern approaches to relational inference employ graph neural networks as their explicit reliance on graph structure forms a relational inductive bias [Battaglia et al. (2016); Battaglia et al. (2018)]. In particular, our model is inspired by the Neural Relational Inference model (NRI) which uses a variational autoencoder for generating edges and a decoder for predicting trajectories of each object in a system [Kipf et al. (2018)]. By inferring edges, the NRI model explicitly captures interactions between objects and leverages the resulting graph as an inductive bias for various machine learning tasks. This model was successfully used to predict the trajectories of coupled Kuramoto oscillators, particles connected by springs, the pick and roll play from basketball, and motion capture visualizations. Subsequently, the authors developed Amortized Causal Discovery, a framework based on the NRI model which infers causal relations from time-dependent data Löwe et al. (2020).

### 2.4 Deep Learning in Neuroscience

With the success of convolutional neural networks, researchers successfully applied deep learning to numerous domains in neuroscience Glaser et al. (2019) including MRI imaging Lundervold and Lundervold (2019) and connectomes Brown and Hamarneh (2016) where algorithms can predict disorders such as autism Brown et al. (2018). Further leveraging the explicit graph structure of neural systems, several studies have successfully applied GNNs on various tasks such as annotating cognitive state Zhang and Bellec, 2019, and several frameworks based on graph neural networks have been proposed for analyzing fMRI data [Li and Duncan (2020); Kim and Ye (2020)].

Similarly, brain-computer interfaces (BCI) are a well-studied field related to our work as they focus on decoding macroscopic variables from measurements of neural activity. These studies generally involve fMRI or EEG data, which characterize neural activity on a population level, to varying amounts of success [Bashivan et al. (2015); Kwak et al. (2017); Mensch et al. (2017); Makin et al. (2020)]. Regardless, a challenge for the field is developing generalizable algorithms to individuals unseen during training Zhang et al. (2019).

## 3 Model

In this section, we first present the general framework of our behavioral state classification and trajectory prediction models. Next, we detail the implementation of our neural network models.

### 3.1 Framework

We define the set of trajectories (calcium imaging traces) for each worm as $X_{\alpha }={\left\{x_{1},\ldots ,x_{n},\ldots ,x_{N}\right\}}_{\alpha }$ where α denotes the label of the individual, *n* the name of the neuron, *N* the total number of neurons, and $x_{n}$ the feature vector of the neuron. In our case, $x_{n}\in {\mathbb{R}}^{T\times 2}$ corresponds to time-dependent normalized calcium traces and their derivatives for each neuron where *T* is the total number of timesteps. Likewise, $x_{n,t}\in {\mathbb{R}}^{2}$ corresponds to the features of neuron *n* at timestep *t*. Finally, the behavioral states of an individual are encoded as $a_{\alpha }={\left(a_{1},\ldots ,a_{t},\ldots ,a_{T}\right)}_{\alpha }$ where a behavioral state *a* is assigned for each timestep *t*.

**FIGURE 3:**
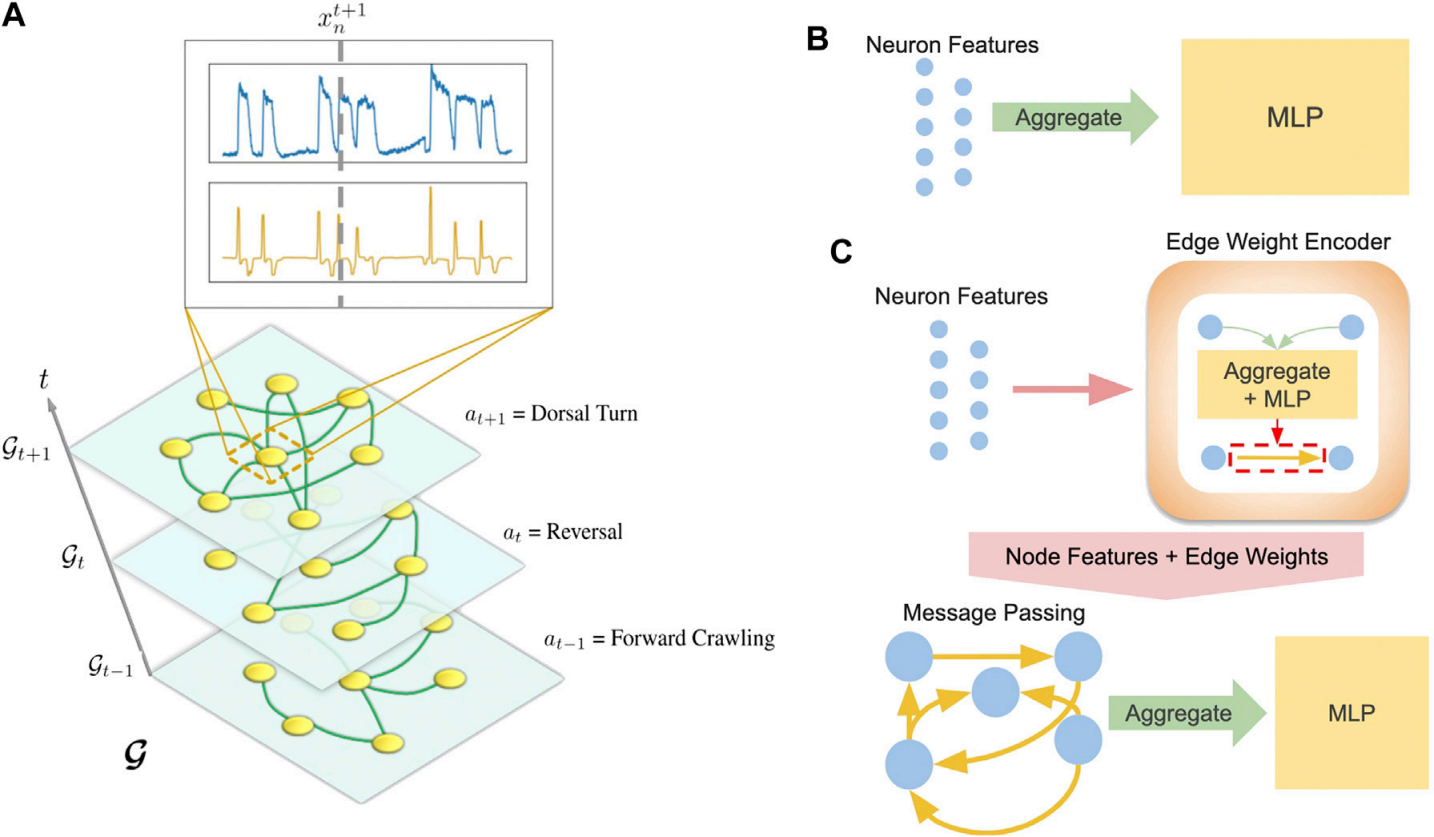
**(A)** Visualization of temporal graph. Inset shows $x_{n}$ plotted against *t* where the top is the calcium trace, and the bottom is its derivative. The dashed line intercepts the feature vectors at t′ = t+1 and denotes $x_{n}^{t+1}$ **(B)** and **(C)** are simplified visualizations of the MLP and GNN models respectively.

**FIGURE 4:**
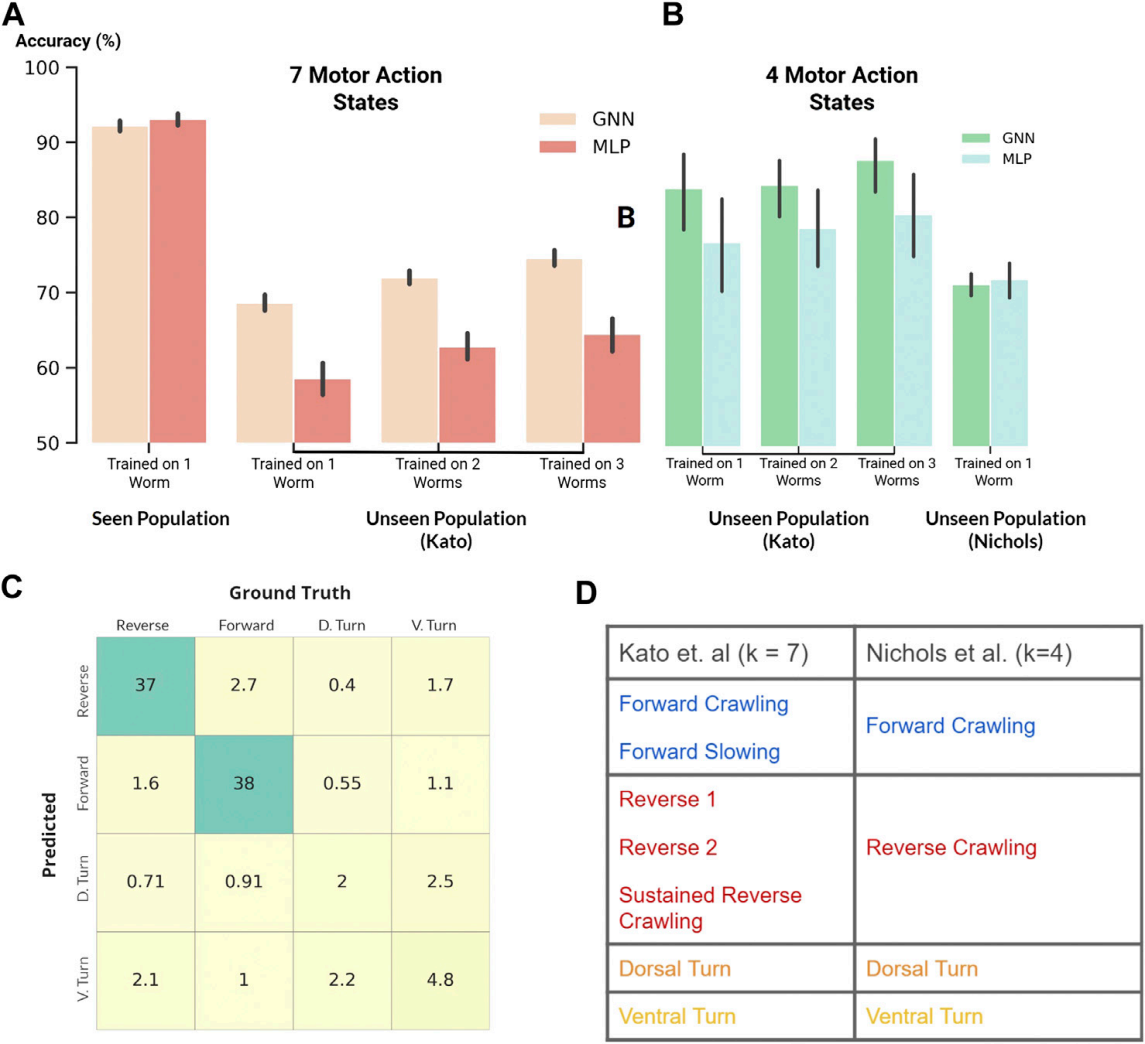
**(A and B)** Classification accuracy of our GNN and MLP models where black vertical lines show statistical spread **(A)**: Classification of seven motor action states within the Kato dataset **(B)**: Classification of four motor action states on both the Kato and Nichols datasets **(C)** Confusion matrix. Percent occurrence of predicted states against labeled states when evaluating on the Nichols dataset **(D)** Mapping of behavioral states between the Kato and Nichols dataset.

Separate models were developed for each task: behavioral state classification and trajectory prediction. In both cases, data from a worm α is structured as a temporal graph $G_{\alpha }={\left(G_{1},\ldots ,G_{t},\ldots ,G_{T}\right)}_{\alpha }$ (Figure 3A) where each timestep is represented by a static graph whose nodes correspond to neurons. Following the notation above for worm α, the trajectories of each neuron’s calcium traces are encoded as node features $x_{n}$, and the behavioral state of the worm is interpreted as a graph feature $a_{t}$. For behavioral state classification, our model consists of the following:

$$H_{\alpha ,t}=f\left(X_{\alpha ,t}\right),$$ (1)

$$p_{\alpha ,t}=softmax\left(H_{\alpha ,t}\right),$$ (2)

$${\hat{a}}_{\alpha ,t}=argmax\left(p_{\alpha ,t}\right).$$ (3)

where $X_{\alpha ,t}$ corresponds to node feature vectors for worm α at timestep *t*, *f* is an universal approximator/neural network model (described in the next section), $H_{\alpha ,t}\in {\mathbb{R}}^{k}$ corresponds to embedded features, $p_{\alpha ,t}$ is the probability that the worm is in one of *k* motor states (Figure 4D), and ${\hat{a}}_{\alpha ,t}$ is the most probable/predicted state.

For trajectory prediction, we developed a Markovian model for inferring trajectories of a consecutive timestep:

$$H_{\alpha ,t}=f\left(X_{\alpha ,t}\right),$$ (4)

$${\hat{X}}_{\alpha ,t+1}=X_{\alpha ,t}+H_{\alpha ,t},$$ (5)

where f is the same as before, $H_{\alpha ,t}$ is the predicted change of the trajectory and can be interpreted as $\text{Δ}{\hat{X}}_{\alpha ,t}$, and ${\hat{X}}_{\alpha ,t+1}$ is the predicted value of the subsequent timestep. When predicting multiple timesteps, the predicted value of the previous timestep is substituted for $X_{\alpha ,t}$. We also experimented with non-Markovian models (RNNs) for which a hidden state is included for each timestep.

The structure of our framework allows us to substitute various models for *f*. While we include results from several neural networks, we focus on two representative models: a multi-layer perceptron (MLP) agnostic to graph structure (Figure 3B) and a graph neural network (GNN) which explicitly computes on an inferred graph (Figure 3C).

### 3.2 Neural Network Models *f*: MLP and GNN

Our MLP model aggregates (sums or concatenates) the features of a graph and feeds the aggregated features into a 2-layer MLP neural network:

$$H_{out}=g_{graph:mlp}\left(\text{aggregation}\left(x_{1},\ldots ,x_{n},\ldots ,x_{N}\right)\right),$$ (6)

where $g_{graph:mlp}$ is a 2-layer MLP. Contrasting the MLP model, our GNN relies on message passing between connected nodes and contains an encoder for edge weights $A_{ij}$:

$$V=g_{node}\left(X\right),$$ (7)

$$E_{ij}=g_{edge}\left(\text{aggregation}\left(v_{i},v_{j}\right)\right),$$ (8)

$$A_{ij}=sigmoid\left(E_{ij}\right),$$ (9)

where in Eq. 7, $V=\left(v_{1},\ldots ,v_{n},\ldots ,v_{N}\right)$ corresponds to the embedding of each node’s features through the MLP $g_{node}$. Next, the edge embedding $E_{ij}$ is computed by aggregating all pairs of node embeddings followed by the MLP $g_{edge}$. Finally, applying the sigmoid function to the edge embedding $E_{ij}$ produces edge weights $A_{ij}$ normalized between 0 and 1. A can be interpreted as an inferred weighted adjacency matrix where $A_{ij}$ denotes the edge weight between nodes i and j such that i=j denotes a self edge. The edge weights either dynamically change in each timestep’s inferred graph $G_{t}$ or remain fixed for the whole temporal graph G of an individual worm. If the edges are static for the temporal graph, the aggregation step in Eq. 8 also averages hidden features across all timesteps such that $V=\frac{1}{T}{\sum^{\text{​}}}_{t=1}^{T}g_{node}\left(X_{t}\right)$. Note that in this case, the edge encoder is given all timesteps $X_{\alpha }$ in Eqs 1, 4 instead of just one timestep.

After edges are encoded, the GNN performs a message passing Eq. 10 and aggregation step Eq. 11:

M=AX, (10)

$$H_{out}=g_{graph:gnn}\left(\text{aggregation}\left(M\right)\right)$$ (11)

As mentioned before, our MLP and GNN models can be subsituted for f in Eqs 1, 4. Depending on the task, the dimension of $H_{out}$ for the MLP Eq. 6 and GNN Eq. 11 models differs. For behavioral state classification, $H_{out}\in {\mathbb{R}}^{k}$ whereas for trajectory prediction, $dim\left(H_{out}\right)=dim\left(X_{\alpha ,t}\right)$ such that $H_{out}\in {\mathbb{R}}^{N\times 2}$.

Theoretically, an arbitrary number of message passing steps can be implemented; however, we did not find any improvements when using more than one step. In addition, we find that performance improves when using concatenation instead of summation during the aggregation step.

## 4 Experiments and Data

Our experiments were performed with data acquired in Kato et al. (2015) and Nichols et al. (2017). We summarize various details about the data in this section; however, we direct the reader to each respective publication for specific experimental details.

### 4.1 Calcium Imaging

Kato et al. (2015) showed that neural activity corresponding to the motor action sequence lives on low dimensional manifolds. To record neuron level dynamics, they performed whole-brain genetically encoded ${\text{Ca}}^{2+}$ imaging with single-cell-resolution and measured ∼100 neurons for around 18 min. They then normalized each calcium trace by peak fluorescence and identified neurons using spatial position and previous literature (Altun et al., 2002–2020). Aside from imaging freely moving worms, the authors also examined robustness of topological features to sensory stimuli changes, hub neuron silencing, and immobilization. For simplicity, we limited our experiments to data collected on freely moving worms.

Nichols et al. (2017) focused on differences in neural activity of *C. elegans* while awake or asleep and studied two different strains of worms, n2 (11 total worms) and npr1 (10 total worms). Because experiments in both studies were performed by the same group, most experimental procedures were similar, allowing us to easily process data to match the Kato dataset. While this dataset includes imaging data of each worm during quiescence, for consistency with the Kato dataset, we only included data before sleep was induced. Furthermore, we pooled results for both strains of worms as we did not notice any statistically relevant differences between them.

### 4.2 Dataset Enlargement

Although our data for each worm is relatively small (∼3,000–4,000 timesteps), our datasets contained calcium traces from numerous worms. In total, 5 worms were measured in Kato et al. (2015) and 21 worms were measured in Nichols et al. (2017). Taking advantage of the large number of worms measured, we experimented with dataset enlargement where our models were trained on pooled data from different numbers of worms in the Kato dataset. Similarly, we pooled data from all 21 worms from the Nichols dataset; however, we use this dataset only during evaluation-i.e., the model never sees this dataset in training. In this way, we define the “seen” population as worms whose data was seen in training and the “unseen” population as worms the model did not see during training. More details about how datasets were used in our experiments can be found in Section 4.2.

To perform dataset enlargement, we separately trained the models on each worm in the seen population for each epoch. In other words, we independently optimized the loss function for each worm in every epoch. We followed this procedure such that batch normalization was separately performed on each worm’s features. This technique was motivated by experiments where batch normalization on data from individual worms improved both test set and generalization accuracy. In contrast, performing batch normalization on pooled data from all worms greatly decreased model performance.

### 4.3 Data Processing

We normalized the calcium trace and its derivative of each neuron to [0,1]. Normalization was performed for the entire recorded calcium trace of a worm instead of within each batch because the relative magnitudes of the traces have been found to contain graded information about the worm’s behavioral state, (e.g. crawling speed).

For the seen population, we separated each calcium trace of approximately 3,000–4,000 timesteps into batches of 8 timesteps where each timestep corresponds to roughly 1/3 of a second. We chose batch sizes of 8 timesteps because visualization of calcium traces showed that most local variations occur within this time frame. Moreover, 8 timesteps roughly corresponds to 3 s which is about the amount of time a worm needs to execute a behavioral change. Finally, the batches were shuffled before being divided into 10 folds later used for cross-validation, ensuring that each fold is representative across the whole dataset.

When evaluating on the unseen population, we treat the data differently for each task. For behavioral classification, we infer the behavioral state of the system using data from one timestep. As such, we do not split the data and simply run the model separately on each timestep of the worm’s calcium traces. In contrast, for trajectory prediction, we split the calcium traces into batches of 16 timesteps and evaluate the model on all batches.

To compare with previous works, we performed our experiments on uniquely identified neurons between the datasets that we investigated. Identifying specific neurons is an experimental challenge, and as such, only a small fraction of neurons were unequivocally labeled. A total of 15 neurons were uniquely identified between all 5 worms measured in the Kato dataset: (AIBL, AIBR, ALA, AVAL, AVAR, AVBL, AVER, RID, RIML, RIMR, RMED, RMEL, RMER, VB01, VB02). In addition, the Nichols dataset contained data from 21 worms with 3 uniquely identified neurons shared among all worms in both datasets: (AIBR, AVAL, VB02).

## 5 Results

Following Brennan and Proekt (2019), we used data from Kato et al. (2015) for training/evaluating our models and data from Nichols et al. (2017) as an extended evaluation set. Because whole brain imaging is incredibly difficult, our datasets were relatively small. To address this, we experimented with dataset enlargement (Section 4.1.2) by combining data from multiple worms in the Kato dataset during model training. For all experiments, we performed 10-fold cross validation on all permutations of worms in our training set. More details, along with supplemental experiments, can be found in the Supplementary Information.

### 5.1 Behavioral State Classification

Our first experiment compared the performance of our models to state-of-the-art results reported in Brennan and Proekt (2019). Specifically, this experiment involved the classification of only two motor action states, forward and reverse crawling. Along with our models described above, we also experimented with a support vector machine (SVM) and a GNN which computes with edges derived from the physical connectome (White et al., 1986). In particular, we incorporated the connectome into our model to investigate whether physical/structural connections between neurons can serve as a favourable inductive bias for our GNN. Our results are shown in Table 1 where “Seen Population” denotes test set accuracy after training on the same worm and “Unseen Population” denotes evaluation/generalization accuracy on worms unseen during training.

**TABLE 1:** Classification accuracy of forward and reverse crawling.

	Seen population	Unseen population (kato)	Unseen population (nichols)
Brennan and Proekt (2019)	83	81	—
SVM	98.8 ± 0.4	82.8 ± 7.6	79.0 ± 11.7
MLP	99.3 ± 0.6	93.9 ± 10.3	88.9 ± 11.4
GNN (connectome)	99.5 ± 0.6	96.8 ± 4.3	85.5 ± 12.9
GNN	99.5 ± 0.5	97.7 ± 3.1	95.5 ± 6.1

Our deep learning models clearly outperformed the SVM and state-of-the-art results, demonstrating the ability of our models to successfully classify behavioral states and generalize to other worms. Interestingly, the SVM matched the performance of our deep learning models on the seen population; however, its generalization performance on unseen individuals was significantly worse than our deep learning models. As such, the SVM distinctly illustrates challenges of individual variability for model development in neural systems despite the simplicity of our experiments which involve the same set of unequivocally identified neurons. Similarly, our GNN using edges derived from the connectome performed well on the seen population but generalized worse than when using inferred edges. We hypothesize that the detrimental effect of using the connectome may be attributed to the distinction between inferred/functional and structural connectivity. In particular, the connectome maps physical connections between neurons which is generally conserved between different individuals. In contrast, individual variability of neural activity implicitly implies that the inferred/functional connectivity is unique to individuals (Supplementary Section S1.4.3).

Following the previous experiment, we applied our MLP and GNN models to the harder task of classifying all behavioral states labeled in the Kato dataset (Figure 4A). Within this dataset, 7 states were labeled: Forward Crawling, Forward Slowing, Reverse 1, Reverse 2, Sustained Reverse Crawling, Dorsal Turn, and Ventral Turn. In comparison to the Kato dataset, only 4 states were labeled in the Nichols dataset: reverse crawling, forward crawling, ventral turn, and dorsal turn. For compatibility, we mapped the 7 states of the Kato dataset to 4 states of the Nichols dataset when using the Nichols dataset as an extended evaluation set (Figure 4D).

Despite the harder task of classifying 7 states, our models achieved a classification accuracy of ∼92% on the same worm (Figure 4A). Moreover, our GNN trained on three worms in the Kato dataset generalized with an accuracy of 87% (Figure 4B) when classifying 4 states on the remaining unseen worms. This substantially exceeds the performance of our MLP model and Brennan and Proekt (2019) who report a 81% cross-animal accuracy on two states. Nevertheless, both MLP and GNN models generalized equally well (∼70%) to the 21 unseen worms of the Nichols dataset. These experiments consistently demonstrate that our GNN exceeds the performance of state-of-the-art techniques and also often exceeds the performance of our baseline MLP model.

### 5.2 Neuron-Level Trajectory Prediction

**FIGURE 5:**
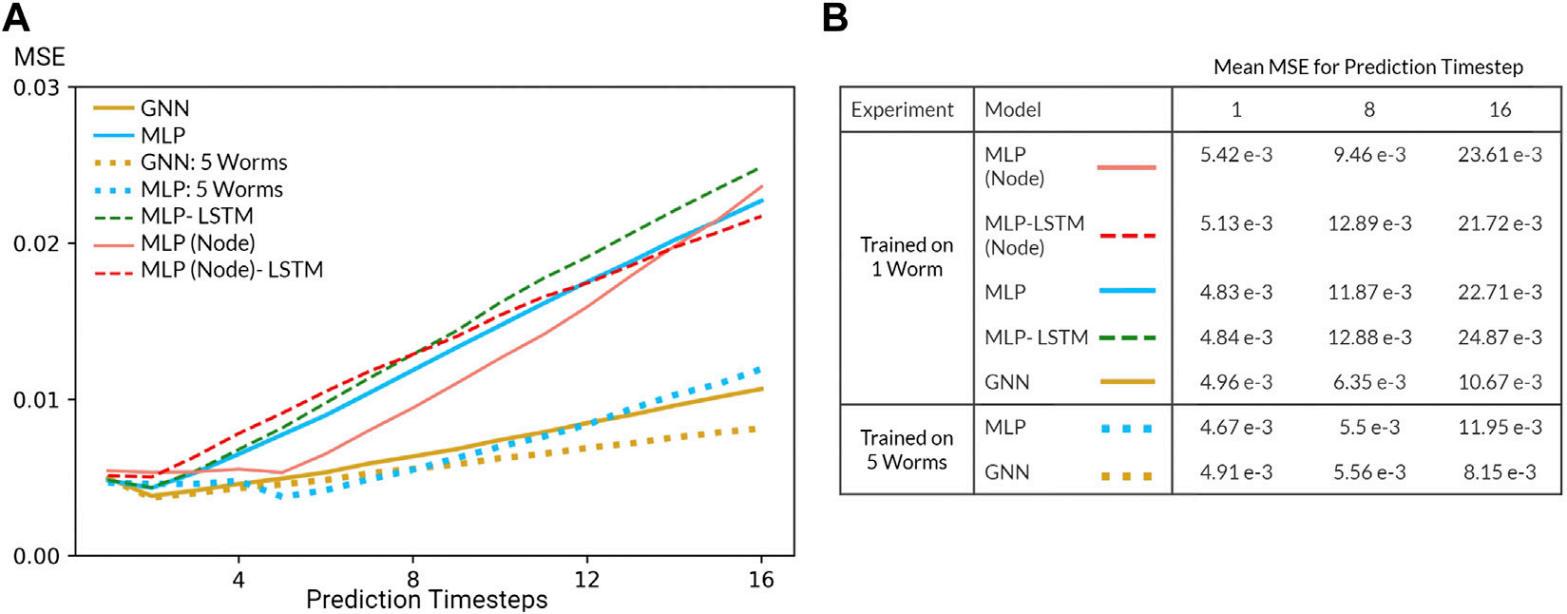
**(A)** Mean squared error (MSE) of the GNN and various MLP models evaluated on the Nichols dataset. All models were trained using data from one worm or five worms in the Kato Dataset **(B)** Table of mean MSE values for all models for 1, 8, and 16 timesteps.

For trajectory prediction, we predicted each neuron’s calcium trace and its derivative (normalized to [0,1]) for 8 timesteps during training (seen population) and 16 timesteps during evaluation/validation (unseen population). While training our Markovian models, scheduled sampling was performed to minimize the accumulation of error (Bengio et al., 2015). When evaluating on the unseen population, the model was given one timestep as the initial condition after which the model predicts 16 timesteps. In addition to our Markovian models, we also experimented with RNN implementations trained with burn-in periods of four timesteps (12 timesteps during training and 20 timesteps during evaluation). Our experiments primarily focused on generalization performance of our models on the extended evaluation/Nichols dataset (Figure 5).

Predicting neuron-level trajectory using deep learning is fairly novel since advances in whole-brain imaging are recent and limited to few organisms. Nevertheless, neural systems generically fall under the category of dynamical systems where each neuron is described by a differential equation such that neural activity can be modeled as a system of coupled differential equations. Under this formulation, the task of trajectory prediction involves learning the underlying physical laws in order to predict the time evolution of the system. To quantify the predictive power of our models, we evaluated the mean squared error (MSE) of each prediction timestep relative to the true trajectory. In the context of our Markovian model, this metric measures the error of the predicted transition matrix which time evolves the state of the system and, by extension, demonstrates the ability of our models to learn the underlying physical laws of the dynamical system.

Several challenges limited the predictive power of our models. Most prominently, our system is inherently non-linear and potentially chaotic, a fact further exasperated by the nature of calcium imaging which is notoriously noisy and an indirect measurement of neural activity. In addition, our datasets are relatively small in spite of our dataset enlargement technique. Resulting from these challenges, the performance of our model is poor, especially in comparison to that of models in data assimilation which leverage a priori knowledge of the dynamical system (). Nevertheless, inspecting the MSE as a function of prediction step (Figure 5) reveals that our models are able to learn how the system transitions up to a short timescale. Moreover, increasing the number of worms included during training (dataset enlargement) also improved generalization performance of our MLP and GNN models. Perhaps most surprising, our Markovian GNN outperformed all MLP models and their derived RNN variants. We attribute this result to the largely deterministic nature of neural dynamics, characterized by sparse bifurcations on the latent manifold, and the inductive bias of GNNs. As a result, given 1 timestep, our GNN outperformed all other models including RNN variants which were given 4 burn-in timesteps. Therefore, we conclude that our GNN displays a favourable inductive bias in contrast to graph-agnostic models on the task of predicting microscopic dynamics.

## 6 Discussion

For both tasks, our GNN consistently matched or exceeded our MLP model which we accredit to its favourable inductive bias. Kato et al. (2015) established that projecting neural dynamics onto three principal components for each worm reveals universal topological structures; however, attempts to project neural dynamics onto shared principal components of all worms failed to display any meaningful structure. Thus, variability in each worm’s neural activity, corresponding to low dimensional manifolds in latent space, is represented by different linear combinations of neurons. In other words, relevant topological structures in latent space are loosely related by linear transformations of node features. We speculate that our GNN’s performance stems from its explicit structure of message passing along inferred edges which is analogous to learning linear transformations of node features (Eq. 10). Based on our experimental results, we further speculate that this inductive bias proves favourable on both microscopic and macroscopic machine learning tasks in neural systems.

Interestingly, our model’s performance was not significantly impacted by using 3 neurons (∼1% of all neurons) instead of 15 (∼5% of all neurons). This is not surprising because neurons strongly coupled to the motor action sequence retain most information (Gao and Ganguli, 2015), a fact consistent with Brennan and Proekt (2019) who found that strategically choosing 1 neuron retains ∼75% of the information contained in the larger set of 15 neurons.

Finally, as a critical question, we ask whether our model’s performance stems from choosing a stereotyped organism that is well studied and biologically simple, or if our results imply a path toward generalizable/universal machine learning in neural systems. While the neurophysiology of *C. elegans* is quite complex, the motor action sequence we studied is relatively simple, especially in comparison to other organisms and cognitive functions. Moreover, organisms are adaptive and capable of learning new behavior, a fact not represented in our dataset. However, a recent astounding study Gallego et al. (2020) measured neural dynamics in monkeys trained to perform action sequences and determined that learned latent dynamics live in low-dimensional manifolds that were conserved throughout the length of the study. By aligning latent dynamics, their model accurately decoded the action of monkeys up to two years after the model was trained despite changes in biology, (e.g. neuron turnover, adaptation to implants). Consequently, we posit that techniques similar to those used in our model may broadly apply to more complex organisms and functions.

## 7 Conclusion

In this study, we examined the ability of neural networks to classify higher-order function and predict neuron level dynamics. In addition, inspired by global organizational principles of behavior discovered in previous studies, we demonstrated the ability of neural networks to generalize to unseen organisms. Specifically, we first showed that our models exceed the performance of previous studies in behavioral state classification of *C. elegans*. Next, we found that a simple MLP performs remarkably well on unseen organisms. Nevertheless, our graph neural network, which explicitly learns linear transformations of node features, matched or exceeded the performance of graph agnostic models in all experiments. These experiments demonstrate that our models are capable of successful evaluation on unseen organisms, both within the same study, and in a separate experiment spaced years apart. Finally, our results show that dataset enlargement through the inclusion of more individuals can significantly improve generalization performance in microscopic neural systems.

We note that our results of generalization on both higher-order functions and neuron-level dynamics (macroscopic and microscopic) suggests wide applicability of our technique to numerous machine learning tasks in neuroscience and hierarchical dynamical systems. A promising research direction is the hierarchical relationship between neuron-level and population-level dynamics. Breakthroughs in this direction may inform machine learning models working with population-level functional and imaging techniques, such as EEG or fMRI, which are readily available and widespread. In addition, in this study, we only focused on simple machine learning tasks and imaging data taken under similar experimental conditions. Further studies may involve more complex tasks such as those involving graded information in neural dynamics, changes in sensory stimuli, acquisition of learned behaviors, and higher-order functions comprised of complicated sequences of behavior. From a machine learning perspective, the development of a recurrent graph neural network for the edge encoder with a suitable attention mechanism may aid model generalization. Additional work is also needed in examining and improving model performance on arbitrary sets of neurons as neuron identification is experimentally challenging and limited to small systems.

## Data Availability

The original data used in the analysis in our paper can be found in original citations and associated repositories on OSF: https://osf.io/2395t/ (Kato et al.); https://osf.io/kbf38/ (Nichols et al.). Additional inquiries can be directed to the corresponding authors.
