# Supplementary material for: Generalizable Machine Learning in Neuroscience Using Graph Neural Networks
Source: Front Artif Intell. 2021 Feb 23;4:618372. doi: 10.3389/frai.2021.618372 (PMC7971515; doi:10.3389/frai.2021.618372)
Supplement: Supplementary file 1 [file datasheet1.pdf]

Supplementary Material

1 MODEL AND EXPERIMENTS

1.1 Model Selection

The two final models included in the main text were chosen for their performance and simplicity. Nevertheless, we experimented with numerous established models which were easily substituted for f. For GNNs, we primarily used the excellent Pytorch Geometric library (Fey and Lenssen, 2019). Tested modules include the GIN-0/GIN-ϵ (Xu et al., 2018), Graph Sage (Hamilton et al., 2017), GAT (Veličković et al., 2018), and Global Attention (Li et al., 2015). In particular, we expected the GIN to outperform the other modules because its expressiveness has been shown to aid transfer learning (Hu et al., 2020); however, because our edges are not explicitly known, we essentially applied the GIN on a fully connected graph. Under this formulation, the GIN-0 simply symmetrizes node features after a message passing step which is similar to the aggregation step of our MLP. We also found that the GIN-ϵ was prone to overfitting. Finally, we tested the GAT which is similar to our model when edges are dynamically inferred each timestep. As a result, we found that the GAT performs equally well on trajectory prediction but performs slightly worse on behavioral state classification.

1.2 Model Implementation

1.2.1 Neural Networks

The two-layer MLP corresponding to g in the main text comprised of linear layers followed by elu activation functions. We also applied batch norm on the output of the two layers. The Node MLP in the main text refers to individual MLPs for each node. To construct RNN variants, we added an LTSM unit before the MLP.

We performed some minor hyperparameter optimization as our combinatorial cross-validation was computationally expensive. Overall, we found our models relatively robust to different hyperparameters. For trajectory prediction, we used hidden layers with 256 dimensions. On the other hand, for behavioral state classification, we used hidden layers with 16 dimensions. Furthermore, we determined that dynamic edges evaluation worked better for trajectory prediction; however, globally evaluated edges for each worm resulted in better performance for behavioral state classification. Finally, for trajectory prediction, we chose to optimize the mean square error (MSE). For behavioral state classification, we optimize the negative log likelihood (NLL).

1.2.2 Support Vector Machine

For the SVM, we used a linear SVM module from sci-kit learn (Linear SVC). Although the SVMs with linear kernels are significantly less expressive than that with non-linear kernels, we chose a linear kernel as our test set accuracy implies that behavioral states are linearly separable. The SVM was trained with the same loss function until a tolerance of 1E-5 was achieved.

1.3 Experimental Procedures

For the extended evaluation set, we chose prelethargus data where 4 states were labeled: reverse, forward, dorsal turn, and ventral turn. For compatibility with the training dataset, we mapped reverse 1, reverse 2,

and sustained reverse crawling to the reverse state. Similarly, we mapped forward crawling and forward slowing to forward. In addition to the 7 or 4 labeled states, there was another labeled state for unknown behavior or quiescence. This state comprised a very small portion of our data, and during training and evaluation, we ignore the result when the target is unknown.

For all experiments in the main text, we perform 10-fold cross validation on all possible permutations of worms in our training set (Kato dataset). For example, on our experiments trained on two worms, the possible permutations of worms are the following: $\{(1, 2), (1, 3), (1, 4), (1, 5), (2, 3), (2, 4), (2, 5), (3, 4), (3, 5), (4, 5)\}$. Experiments labeled with ”Train on 2 worms” involved models trained separately on each of these permutations. Each permutation then involved 10-fold cross validation where the test set was left out when performing hyperparamter optimization. In particular, for our experiments on behavioral state classification, we used 1 fold as the test/”leave-out” set and 1 fold for the validation set which was used for optimization and as a metric for stopping training. On the other hand, our experiments on trajectory prediction was focused primarily on generalization performance instead of test set accuracy so we used 1 fold as the validation set and evaluated on all worms in the extended validation set (Nichols dataset). As a note, we also attempted experiments where data from the extended dataset was used as a validation set. Under this condition, we found that the MLP performed significantly better; however, we were concerned that the MLP was overfitting to the validation set so we chose not to included those results.

We performed our experiments on with an Intel i9 9900k CPU and Nvidia GeForce RTX 2080Ti graphics card. Since our models are relatively simple, we were able to train the model on data from one worm in one batch. Nevertheless, the number of worms and cross-validation procedure was very computationally expensive. As such, training and evaluating each model required roughly a week or two of continuous computation. For optimization, we used the Adams optimizer with a learning rate of 10^{-3}. We decayed the learning rate with by a factor of .25 if the loss did not improve after 50 epochs. We then trained for 800 epoch and saved the model with the lowest validation loss. For scheduled sampling (used during trajectory prediction), we adopted a linear decay which terminated at 300 timesteps.

1.4 Additional Experiments

We performed numerous experiments to verify our results and examine the performance of our model on diverse machine learning tasks. We did not perform rigorous cross validation for the following experiments.

1.4.1 Experiments without AVA

Referees of Brennan and Proekt (2019) were concerned with behavioral state classification where AVA neurons were included. In particular, these neurons were used by Kato et al. (2015) to define behavioral state through trajectory clustering in latent space. Referees commented that classifying behavioral states with neurons used to define those states was akin to circular reasoning. We would like to note that Kato et al. (2015) verified their assigned behavioral states through recorded videos, minimizing risks that assigned behavioural states differ from reality. Nevertheless, we followed Brennan and Proekt (2019) and performed an experiment excluding AVA neurons in which we found no noticeable difference in model performance.

1.4.2 One-hot encoding of edges

To enforce a sparsity on the edges, we experimented with one-hot encoding by adding a scaling factor within the softmax. We found that our GNN achieved similar test accuracies as in the main text. However, our GNN failed to generalize well to unseen worms. Following our discussion in the main text, we believe that one-hot encoding was detrimental to generalization because it effectively results in a permutation matrix

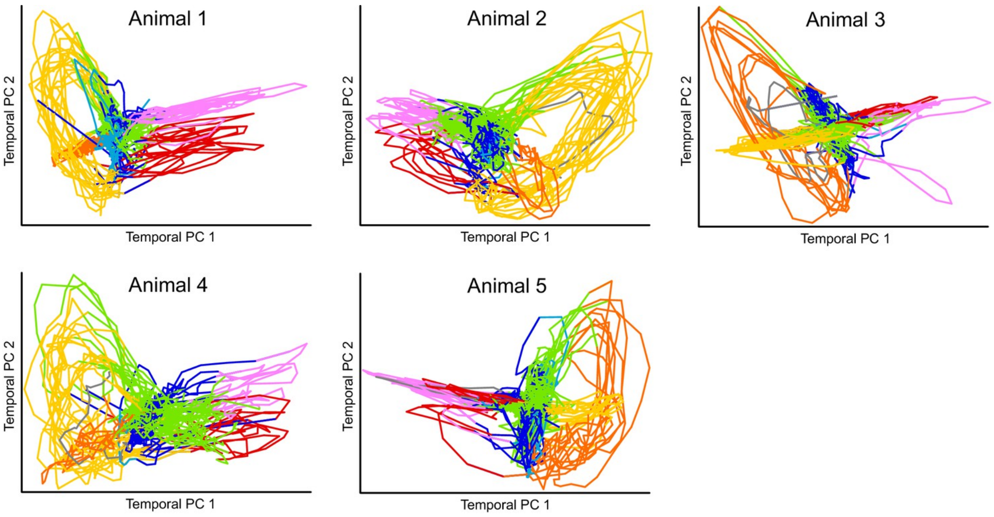

Figure S1. Time derivatives of calcium traces projected onto each individual organism's principal components. Distinct loops correspond to manifolds in latent space where colors correspond to behavior assigned in Kato et al. Reproduced with permission from Brennan and Proekt (2019).

which simply permutes node features. This is counter to previous studies where topological structures are related by more general linear transformations.

1.4.3 Comparison of inferred edges to known connectome

Inferring the connectivity between neurons in neural systems remains a key challenge in neuroscience. Because C. Elegans is among few organisms whose connectome mostly or completely known, we decided to compare the inferred edges of our model to the connectome of C. Elegans. Ultimately, we found no similarities between our inferred edges and the connectome.

In neuroscience, two types of connectivity are defined: structural and functional. Structural connectivity refers to physical connections between neurons whereas functional/effective connectivity corresponds to observed connections (Horwitz, 2003). The exact methods for determining either metrics remains heavily contested. Regardless, in the context of C. Elegans, each worm generally has the same structural connectivity; however, differences in neural activity implies a different functional connectivity exists for unique individuals. Since the connectome relates to the structural connectivity, we believe that our inferred edges are a poor proxy for the connectome. On a more abstract level, our graph neural network works with a subset of neurons such that a inferred edge may not correspond to a direct correlation, but may rather represent higher order correlations with unseen neurons.

REFERENCES

Brennan, C. and Proekt, A. (2019). A quantitative model of conserved macroscopic dynamics predicts future motor commands. *Elife* 8, e46814

Fey, M. and Lenssen, J. E. (2019). Fast graph representation learning with PyTorch Geometric. In *ICLR Workshop on Representation Learning on Graphs and Manifolds*

Hamilton, W., Ying, Z., and Leskovec, J. (2017). Inductive representation learning on large graphs. In *Advances in neural information processing systems*. 1024–1034

Horwitz, B. (2003). The elusive concept of brain connectivity. *Neuroimage* 19, 466–470

Hu, W., Liu, B., Gomes, J., Zitnik, M., Liang, P., Pande, V., et al. (2020). Strategies for pre-training graph neural networks

Kato, S., Kaplan, H. S., Schrödel, T., Skora, S., Lindsay, T. H., Yemini, E., et al. (2015). Global brain dynamics embed the motor command sequence of caenorhabditis elegans. *Cell* 163, 656–669

Li, Y., Tarlow, D., Brockschmidt, M., and Zemel, R. (2015). Gated graph sequence neural networks. *arXiv preprint arXiv:1511.05493* `https://arxiv.org/abs/1511.05493`

Veličković, P., Cucurull, G., Casanova, A., Romero, A., Lio, P., and Bengio, Y. (2018). Graph attention networks

Xu, K., Hu, W., Leskovec, J., and Jegelka, S. (2018). How powerful are graph neural networks? *International Conference on Learning Representations*
