# Supplementary figures and images for: Generalizable Machine Learning in Neuroscience Using Graph Neural Networks
Source: Front Artif Intell. 2021 Feb 23;4:618372. doi: 10.3389/frai.2021.618372 (PMC7971515; doi:10.3389/frai.2021.618372)

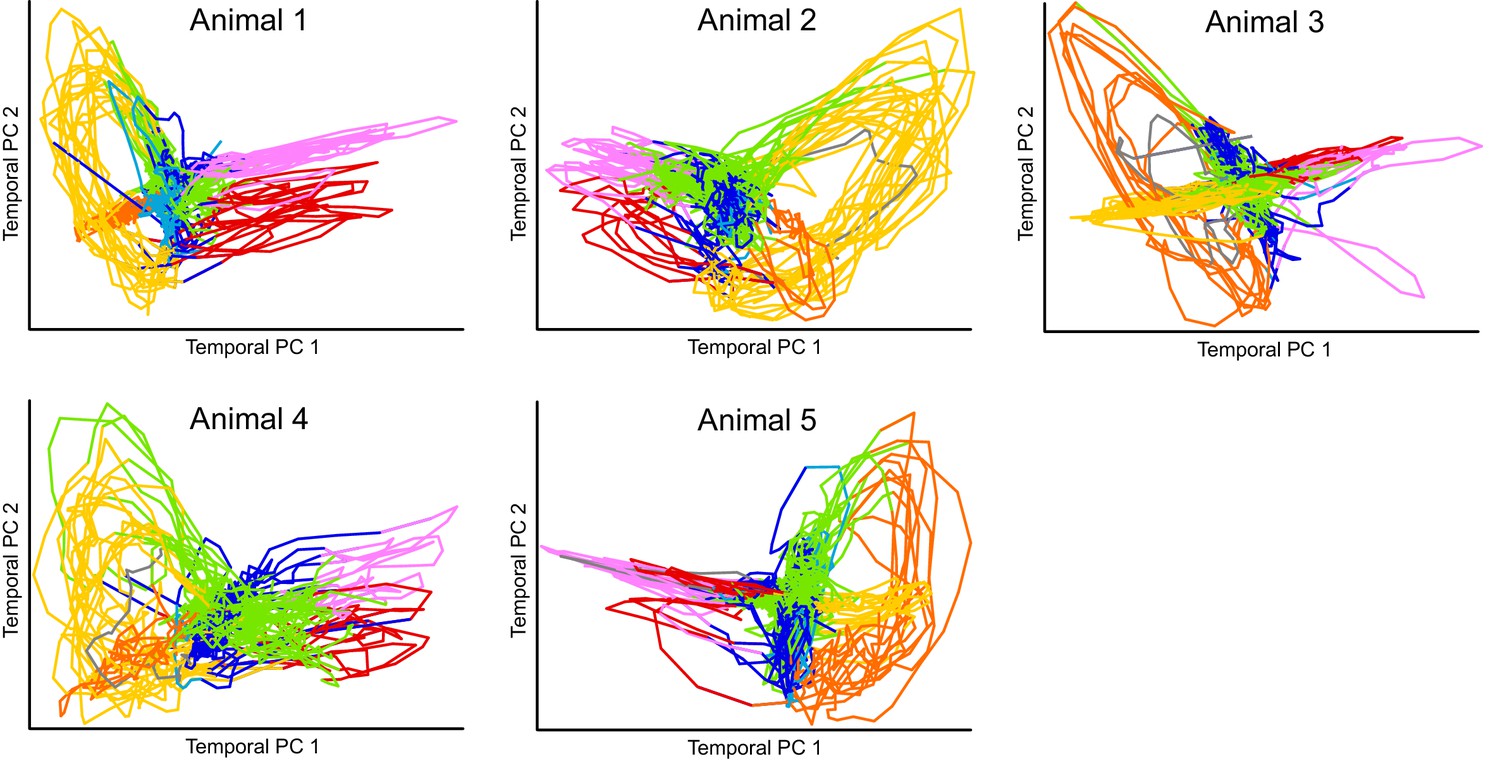

Supplement: Supplementary file 2 [file image1.jpeg]
